# Blindness and Visual Impairment among Egyptian Glaucoma Patients

**DOI:** 10.1155/2014/437548

**Published:** 2014-01-29

**Authors:** M. A. Eldaly, M. M. Salama, K. G. Abu Eleinen, D. Ghalwash, M. Youssef, A. F. El-Shiaty

**Affiliations:** Ophthalmology Department, Faculty of Medicine, Cairo University, Al-Saray Street, El Manial, Cairo 11956, Egypt

## Abstract

*Purpose*. Glaucoma is the second commonest cause of blindness worldwide. Visual fields and intraocular pressures are optimum outcomes to be assessed in developed countries. Visual acuity as an outcome is the key player when assessing blindness in developing countries. The aim of this study is to assess visual impairment and blindness in glaucoma patients and to identify the bulk they represent in comparison to nonglaucoma patients in the same practice setting. *Methods*. Patients attending outpatient clinics of Cairo University Hospitals were enrolled in this cross-sectional study. Clinical data collected for these patients included their demographic data, best obtained visual acuity, and whether or not they have had an established diagnosis of glaucoma. Results were compared at 95% confidence intervals. *Results*. 988 eyes of 494 patients were included for this study. Their mean visual acuity was 6/24 (±3 Snellen lines). Legal blindness was found in 5% and 4% of glaucoma and nonglaucoma groups, respectively. There were high odds for finding eyes with total blindness due to glaucoma. That was statistically significant (*P* < 0.05). *Conclusions*. A large proportion of glaucoma patients suffered from blindness in at least one eye. Interventional strategies are recommended regarding visual disability among Egyptian glaucoma patients.

## 1. Introduction

The World Health Organization (WHO) estimated in a systemic review that glaucoma is the second commonest cause of blindness worldwide [1]. In spite of this data, glaucoma was not included in the top priority five diseases for the first phase of the Vision 2020 program of Africa [2]. This could be partly explained by the uncertainties that have existed concerning the evidence for case detection [3]. With the scarcity of population-based surveys, clinic-based ones provide important data for health care delivery in ophthalmic practices. Many studies have assessed the clinical impact of glaucoma as regards visual fields and intraocular pressure as primary outcomes. This is optimum for developed countries but for developing countries, visual acuity as a primary outcome of assessment for glaucoma needs to be also studied. The aim of this clinic-based study is to assess patterns of visual acuity and blindness in glaucoma patients and to find out the bulk they represent in comparison to nonglaucoma patients in the same practice setting.

## 2. Methods

Patients attending Cairo University Hospital's ophthalmic clinics were enrolled in this cross-sectional clinic-based study. Inclusion criteria included all patients attending the ophthalmic outpatient clinic for one day of work, only one clinic for each unit, to avoid detection bias (ophthalmology departments consist of subunits with each unit running separate clinics). Tenets of Helsinki Declaration and the World Medical Association Declaration on ethical considerations regarding health databases were respected. Clinical data collected for these patients included their demographic data, best obtained visual acuity, and whether or not they have had an established diagnosis of glaucoma. Ophthalmologists who collected the data did not participate in data analysis nor their interpretation to avoid performance bias.

In this study, legal blindness was defined as visual acuity worse than 3/60 in the better seeing eye irrespective of the status of the visual field. Visual impairment was defined as visual acuity 3/60 or better but worse than 6/18 in the better seeing eye irrespective of the status of the visual field. A totally blind eye was defined as an eye with vision of hand motions or less. Legal blindness and visual impairment were assessed per patient while totally blind eyes were assessed per eye. The definitions of legal blindness and visual impairment were guided by the Egyptian law for the measurement of visual disability, which is exclusively dependent on the visual acuity to identify the degree of disability [4]. The primary outcome from this study was to identify the levels of visual acuity affection in glaucoma patients in comparison to visual acuity of those without glaucoma in the same practice setting. The secondary outcome was to know the prevalence of glaucoma patients in this practice setting. Data were tabulated and analyzed. Statistical analysis was done using SPSS software (SPSS Inc., Chicago, IL, USA). Statistical significance tests were at the 5% probability level.

## 3. Results

**Figure 1:**
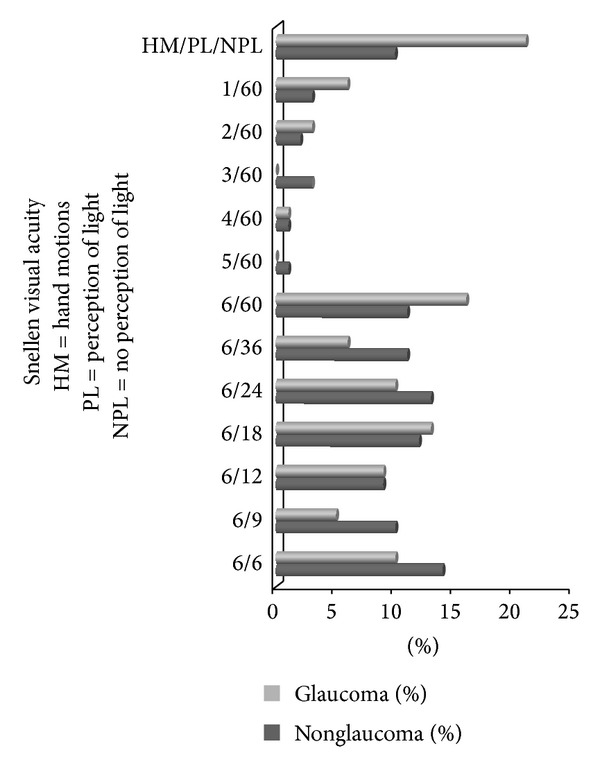
Patterns of visual acuity in an Egyptian ophthalmic Clinic.

**Figure 2:**
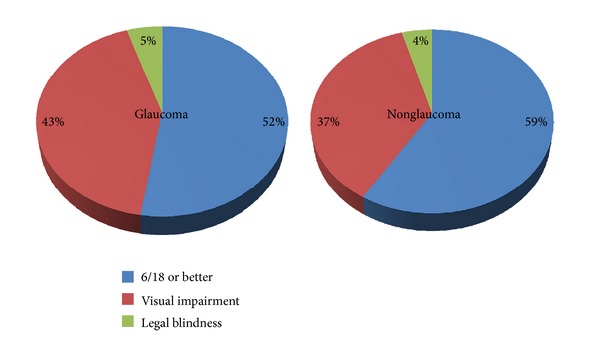
Legal blindness and visual impairment assessed per patient.

Nine hundred and eighty-eight eyes of 494 patients who had the mean age of 44.02 (SD 18.8) years were enrolled in this survey. Female to male ratio was 1.2 : 1 and their mean visual acuity was similar in either eye, 6/24 (±3 Snellen lines). The prevalence of patients with glaucoma was 8.1% (confidence; level 95%, interval 2.1–4.1). The differences between glaucoma and nonglaucoma groups as regards age and sex were not statistically significant. Similar was the visual acuity of either eye when compared collectively and subgrouped. Patterns of visual acuities for glaucoma patients and nonglaucoma ones are shown in Figure 1. The weights of visual impairment and legal blindness when assessed per patient between either groups were not statistically significant (*P* = 0.074) (Figure 2). When analyzing eyes with total blindness, glaucoma ones had shown high odds of getting total blind eyes and that was statistically significant (Table 1).

## 4. Discussion

One percent of Africans are blind in whom glaucoma is the leading cause of irreversible blindness [5, 6]. The earliest population-based survey for the patterns of visual acuity and blindness in Egypt was done between 1965 and 1968. They reported that the prevalence of patients with vision 1/60 or less were 16.7% higher in rural areas when compared to the urban areas, but they did not report the rates of legal blindness nor total blind eyes from glaucoma. However, we can find in their published tables that the prevalence of glaucoma was 2% in urban population and 9% in the rural one [7]. Similarly, we found a prevalence of 8% in our clinic-based study. Few regional clinic-based reports have evaluated blindness of glaucoma in Egypt. In these surveys from different Egyptian cities, the prevalence of blindness from glaucoma was 12.1% at Mansoura in 2002, 19.7% at Alexandria in 1987, and 7.6% at Al-Azhar university study (Cairo) in 1989 [8–10]. Similarly, the proportion of blindness from glaucoma in clinic-based surveys from different African countries ranged from 10% to 33% [3]. In a hospital-based study in Nigeria, researchers reported that 23.1% of blind patients were due to glaucoma [11]. We found highly significant odds for recording eyes with total blindness in eyes with glaucoma (30%). In a hospital-based study in Addis Ababa, the investigators detected even higher rates for blind eyes from glaucoma (44%) [12]. This could be either due to a delay in presentation, diagnosis, or many other factors. Eldaly et al. asserted that multiple factors affect status of glaucoma patients [13].

The definitions of blindness at which visual acuities were assessed were not uniform among the previous studies. In the current report which was done in Cairo, the better figures of legal blindness rates compared to earlier studies, either reflects improvements in glaucoma health care or was underestimated because we did not include visual fields in our legal blindness assessment, which would have increased those who were legally blind. Amending the national laws [4] and considering the status of visual fields when assessing the visual disability is recommended to fulfill the requirements advised by the WHO regarding legal blindness and visual impairment. The high rates of the totally blind eyes found for glaucoma in this study mandates the importance to investigate the factors behind these figures on a wider scale. This necessitates the need for interventional strategies to manage the problem of glaucoma among Egyptian patients.

## Figures and Tables

**Table 1:** Relative risk for glaucoma eyes presenting with total blindness.

	Rate	Risk ratio	Confidence interval		Odds	Odd ratio	Confidence interval		*P* value
			Upper	Lower			Upper	Lower	
Glaucoma	0.3	1.8	1.3	2.7	0.4	2.2	1.3	3.7	<0.05
Nonglaucoma	1.6				0.2				
